# Isolation, characterisation and complement fixation activity of acidic polysaccharides from *Argemone mexicana* used as antimalarials in Mali

**DOI:** 10.1080/13880209.2022.2089691

**Published:** 2022-07-07

**Authors:** Adama Dénou, Adiaratou Togola, Kari Tvete Inngjerdingen, Nastaran Moussavi, Frode Rise, Yuan Feng Zou, Dalen G. Dafam, Elijah I. Nep, Abubakar Ahmed, Taiwo E. Alemika, Drissa Diallo, Rokia Sanogo, Berit Smestad Paulsen

**Affiliations:** a Department of Pharmaceutical Sciences, Faculty of Pharmacy, University of Sciences, Techniques and Technologies of Bamako, Bamako, Mali; b Department of Pharmacognosy and Traditional Medicine, Faculty of Pharmaceutical Sciences, University of Jos, Jos, Nigeria; c Section for Pharmaceutical Chemistry, Department of Pharmacy, University of Oslo, Oslo, Norway; d Department of Chemistry, University of Oslo, Oslo, Norway; e Natural Medicine Research Center, College of Veterinary Medicine, Sichuan Agricultural University, Chengdu, P.R. China; f Department of Pharmaceutical Chemistry, Faculty of Pharmaceutical Sciences, University of Jos, Jos, Nigeria

**Keywords:** Argemone Mexicana, polysaccharides, complement system, immunomodulation, Mali

## Abstract

**Context:**

Global studies on *Argemone mexicana* L. (Papaveraceae) traditionally used against malaria in Mali are limited to its low-mass compounds activities, and little information on its bioactive polysaccharides is available.

**Objective:**

This study determines the structure and the immunomodulatory activity of polysaccharides from aerial parts of *A. mexicana*.

**Materials and methods:**

Acidic polysaccharides from this plant material named HMAmA1 and HMAmA2 were isolated from water extracts. Their monosaccharide composition was determined by gas chromatography. Glycosidic linkages were determined using GC-MS. NMR was also applied. The polymers were tested for effects on the human complement system *in vitro* at different doses.

**Results:**

The monosaccharide composition showed that the two polysaccharides contained in different amounts the following monomers: arabinose, rhamnose, galactose, and galacturonic acid. Overall structural analysis showed the presence of a low ratio of 1,2-linked rhamnose compared to 1,4-linked galacturonic acid with arabinogalactans substituted on position 4 of rhamnose. NMR data showed the presence of galacturonans alternated by rhamnogalacturonans bearing arabinose and galactose units. α-Linkages were found for l-arabinose, l-rhamnose and d-galacturonic acid, while β-linkages were found for d-galactose. The two polysaccharides exhibited strong complement fixation activities, with HMAmA1 being the highest potent fraction. ICH_50_ value of HMAmA1 was 5 µg/mL, compared to the control BPII being 15.9 µg/mL.

**Discussion and conclusions:**

Polysaccharides form *A. mexicana* presented a complement fixation effect. The complement system is an important part of the immune defense, and compounds acting on the cascade are of interest. Therefore, these polymers may be useful as immunodulatory agents.

## Introduction

*Argemone mexicana* L. (Papaveraceae) is an herbal plant with prickly spikes both on its greenish stem and the pinnate lobed leaves; the flower is terminal and yellow and the fruit is a capsule with thorns (Dénou et al. 2020a). *Argemone mexicana* is indigenous in Mexico and the West Indies but has become pantropical after accidental introduction or introduction as an ornamental. It is naturalised in most African countries, from Cape Verde east to Somalia, and south to South Africa according to Bosch in 2008 as reported by Thorat and Ghorpade (2018). In Mali, the aqueous aerial part extract of this plant is used traditionally against malaria (Diallo et al. 2007). Several pharmacological activities including antimicrobial, anti-HIV, anti-inflammatory, wound healing, anti-stress, anti-allergic, vasoconstrictor and vasorelaxant effect, antifertility, cytotoxic, nematicidal, anti-feeding effect on ileum organ, fungi toxic, antioxidant, anticancer, antidiabetic, antihepatotoxic and many more miscellaneous properties were reported from this plant species (Sharanappa and Vidyasagar 2014; Ibrahim et al. 2016; Husna and Reddy 2017; Pathak et al. 2021). Phytochemicals reported are carotenoids, phenolics, alkaloids, pectins, tannins, coumarins, flavonoids, amino acids, saponins and terpenoids (Sanogo et al. 2014, Sharanappa and Vidyasagar 2014; Ibrahim et al. 2016; Dénou et al. 2020a; Pathak et al. 2021). To date, there are very few reports on polysaccharides from *Argemone mexicana* (Dénou et al. 2019), and thus it would be of interest to investigate the structural properties and immunomodulatory activity of its polysaccharides.

Polysaccharides are biomacromolecules consisting of carbohydrate molecules linked to each other through glycosidic bonds. These polymers play an important role in the development of pharmaceuticals, food, nutritional products, and biodegradable packaging materials (Copeland et al. 2009; Yang and Zhang 2009; Cazón et al. 2017; Yu et al. 2018). They exhibit a variety of biological activities such as immunomodulatory, antioxidant, antiaging, antitumor and anti-inflammatory activities (Paulsen and Barsett 2005; Ray et al. 2020). Polysaccharides are found in plants, fruits, vegetables, herbs, algae, mushrooms, and microorganisms. To extract these polymers water is commonly used as the solvent. However hot water has been found to be a better extracting solvent than cold water (Nemzer et al. 2019). Polysaccharides can be linear or branched, and they can be classified as homo-polymers if the polymer is composed of identical monosaccharides, or heteropolymers if the polysaccharide is composed of two or more different monosaccharides (Nemzer et al. 2019). As such, the structures of these polymers can be determined using various sophisticated analytical and spectroscopic techniques, including Fourier transform infra-red spectroscopy (FT-IR), ultraviolet spectrophotometry (UV-Vis), nuclear magnetic resonance (NMR) spectrophotometry, gas chromatography-mass spectrophotometry (GC-MS), and liquid chromatography-mass spectrophotometry (LC-MS). It has also been found that their structural properties vary based on the natural sources, processing methods, extraction methods, and variety of agricultural or botanical species (Cui 2005; Wang et al. 2013; 2016). Plant polysaccharides have been shown to exhibit biological effects related to the immune system by different *in vitro* assays (Grønhaug et al. 2010).

The complement system cascade is an important part of the innate immune defense. Proteolytic cleavage of complement components by activation of one or more of its three pathways leads to the generation of complement activation products. These mediators exert many biological activities such as the increment of local vascular permeability, the attraction of leucocytes (chemotaxis), immune adherence and modulation of antibody production. Hence, activation of the complement system will contribute to inflammatory responses and immunological defense reactions. The interaction with the complement system by polysaccharides due to fixation could be a good therapeutic strategy for treating inflammatory diseases (Yamada and Kiyohara 1999). Investigation of polysaccharides with complement fixation activity could lead to the discovery of potential immunomodulators.

Therefore, this study describes the isolation, characterisation, and complement fixation activity of polysaccharides from 100 °C water aerial part extract of *A. mexicana*. The water extract was purified by ANX Sepharose™ 4 Fast Flow anion exchange chromatography, and gas chromatography, gas chromatography-mass spectrometry (GC-MS), and nuclear magnetic resonance was employed for their structural elucidation. The complement fixation activity of the analysed polysaccharides was also evaluated.

## Material and methods

### Chemicals

Dichloromethane, ethanol 96%, distilled water, ANX Sepharose™ 4 Fast Flow, NaCl, 4% phenol in sulphuric acid, 3 M hydrochloric acid in methanol, mannitol, veronal buffer, bovine serum albumin and 0.02% sodium azide were all of the analytical grade and purchased from Sigma Aldrich, St. Louis, MO, USA.

### Plant material

The aerial parts of *Argemone mexicana* were collected at Blendio in Mali, in September 2014, and identified at the Department of Traditional Medicine (DMT) by Late Professor Drissa Diallo. A voucher specimen is preserved at the herbarium of DMT (Voucher No. 2948/DMT) for future reference. The plant material was dried under shade at room temperature for two weeks then it was pulverised to a fine powder using a mechanical grinder.

### Extraction and isolation of acidic polysaccharides

The plant material was extracted by the procedure given below starting with the most lipophilic solution and followed with a lesser degree of lipophilicity and last with water. Briefly Accelerated Solvent Extraction was performed on a Dionex ASE 350 Accelerated Solvent Extractor (Dionex, Sunnyvale, CA, USA). Powdered aerial parts (500 g) were weighed and mixed with 125 g of diatomaceous earth. 316.5 g of the mixture were packed in eight stainless steel cells of 100 mL repeated two times. The extractions were performed at 1,500 psi, with 5 min heating, 5 min static time, and a 250 s purge for a total of three cycles.

**Figure 1.**
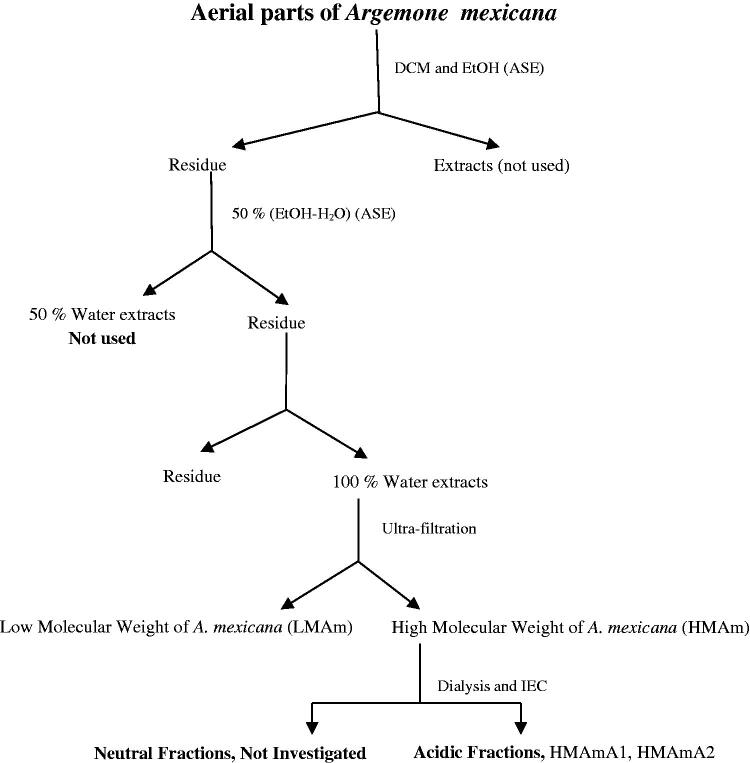
Scheme of the extraction and fractionation of polysaccharides from aerial parts of *Argemone mexicana,* using accelerated solvent extraction (ASE), ultrafiltration and ion exchange chromatography (IEC).

In order to remove low molecular weight compounds including lipophilic components, the cells were subjected to pre-extraction three times with dichloromethane (DCM) at 40 °C, followed by 96% EtOH (ethanol) at 60 °C. The residues in the cells were further extracted three times with 50% EtOH-H_2_O (ethanol-water) at 50 °C, followed by distilled water at 100 °C two times. Then the 50% EtOH-H_2_O extract was concentrated and lyophilised while the water extracts were gathered and subjected to ultrafiltration (cut off 5,000 Da). The low molecular weight (LMAm) fraction was concentrated and lyophilised whilst its high molecular weight (HMAm) fraction was dialysed at a cut-off of 3,500 Da. The extraction and fractionation of *A. mexicana* are shown in Figure 1. The dialysed high molecular weight fraction was used to obtain the polysaccharide fractions by ion exchange chromatography.

The dialysed high molecular weight fraction from Accelerated Solvent Extraction (ASE) was applied to an anion exchange column (XK50) packed with ANX Sepharose™ 4 Fast Flow (high sub) (GE Healthcare, Uppsala, Sweden). The neutral fractions were eluted with distilled water at (1 mL/min), while the acidic fractions were eluted with a linear NaCl gradient in water (0–1.56 M) at 2 mL/min. The carbohydrate elution profiles were monitored using the phenol-sulphuric acid method (DuBois et al. 1956). The related fractions were pooled, dialysed at cut-off 3500 Da against distilled water for removal of NaCl, and lyophilised prior to characterisation. For quality assurance issues these polymers were subjected to a safety evaluation in *Drosophila melanogaster* as previously described (Dénou et al. 2020b).

### Characterisation of acidic polysaccharides

The monosaccharide compositions of the fractions were determined by gas chromatography of the trimethylsilylated (TMS) derivatives of the methyl-glycosides obtained after methanolysis with 3 M hydrochloric acid in anhydrous methanol for 24 h at 80 °C (Chambers and Clamp 1971; Barsett et al. 1992; Austarheim et al. 2012) (1 mg sample in 1 mL of 3 M hydrochloric acid in anhydrous methanol). Mannitol (100 µg) was used as an internal standard. The TMS derivatives were analysed by capillary gas chromatography on a Focus GC (Thermo Scientific, Milan, Italy).

Glycosidic linkage elucidation of the polymers was performed by ethylation studies. Prior to ethylation, the free uronic acids were reduced with NaBD_4_ to their corresponding neutral sugars. After the reduction of the polymers, ethylation, hydrolysis, reduction and acetylation (Kim and Carpita 1992) were carried out using amounts as described in the reference. The derivatives were analysed by GC-MS using a GCMS-QP2010 (Shimadzu, Kyoto, Japan) attached to a Restek Rxi-5MS (30 m; 0.25 mm i.d.; 0.25 µm film) column. The injector temperature was 280 °C, the ion source temperature was 200 °C and the interface temperature 300 °C. The column temperature was 80 °C when injected, then increased by 10 °C/min to 140 °C, followed by 4 °C/min to 210 °C and then 20 °C/min to 300 °C. Helium was the carrier gas (pressure control: 80 kPa). The compound at each peak was characterised by an interpretation of the retention times and the characteristic mass spectra. The estimation of the relative amounts of each linkage type was related to the total amount of each monosaccharide type as determined by methanolysis. Effective carbon-response factors were applied for quantification (Sweet et al. 1975).

^1^H NMR, ^13^C NMR HMBC and HSQC spectra of the two polysaccharide fractions (HMAmA1 and HMAmA2) were obtained on an 800 NMR-spectrometer (800.03 MHz) after deuterium was exchanged three times by freeze-drying in D_2_O. ^1^H NMR spectra with solvent suppression, ^13^C NMR and HSQC spectra with shaped decoupling pulses and solvent suppression of the HOD line were recorded in D_2_O solution on a Bruker AVIIIHD 800 instrument (Bruker, Fällanden, Switzerland) at a temperature of 60 °C.

### Complement fixation activity

The complement fixation test is based on the inhibition of haemolysis of antibody sensitised sheep red blood cells (SRBC) by human sera as described by Michaelsen et al. (2000). It is a quick, highly reproducible assay performed using microtiter plates with many samples analysed simultaneously and with positive control. Sheep erythrocytes were washed twice with 9 mg/mL NaCl and once with veronal buffer pH 7.2 containing 2 mg/mL bovine serum albumin (BSA) and 0.02% sodium azide (VB/BSA) and sensitised with rabbit anti-sheep erythrocyte antibodies (Viron amboceptor 9020, Ruschlikon, Switzerland). After incubation at 37 °C for 30 min on a shaker, the cells were washed as described above, and a 1% cell suspension in veronal buffer was prepared. The serum was diluted with VB/BSA to a concentration giving about 50% haemolysis. Samples dissolved in VB/BSA (250, 62.5, 15.6, 3.9, 0.98 and 0.25 µg/mL) (50 mL) and serum (50 mL) were added in duplicate to wells on a microtiter plate and incubated on a shaker at 37 °C. After 30 min, the sensitised sheep erythrocytes (50 µL) were added and the microtiter plate was incubated as earlier. After centrifugation at 1000 × *g* for 5 min, 100 µL of the supernatants were transferred to a flat bottom microtiter plate and the absorbance at 405 nm was measured using a microtiter plate reader. 100% lysis was obtained with distilled water and sensitised sheep erythrocytes (=A_water_). The control of the medium was VB/BSA, serum and sensitised sheep erythrocytes (= A_control_). BPII, a highly active pectic polysaccharide from the aerial parts of *Biophytum petersianum* Klotzsch (Oxalidaceae) (syn. of *B. umbraculum*) (Grønhaug et al. 2011), was used as a positive control. The indicator system in the assay is the inhibition of haemolysis induced by human complement. Samples showing inhibition in the assay are thus having a direct effect on the human immune system. Each acidic polysaccharide fraction and BPII was tested at different concentrations from 0.2-250 µg/mL. The inhibition of lysis induced by the test samples was calculated by the formula:

$$\text{Inhibition of lysis}=(A_{\mathrm{control}}-A_{\mathrm{sample}})/A_{\mathrm{control}}\times 100$$

where *A*_control_ is the absorbance of control; *A*_sample_ is the absorbance of the sample.

From these data, a dose-response curve was constructed and the concentration of the test sample giving 50% inhibition of lysis (ICH_50_) was calculated. A low ICH_50_ value means a high complement fixing activity. This biological test system can have some day-to-day variation and thus the ratio ICH_50_ BPII/ICH_50_ for each sample was calculated. A high ratio means high complement fixing activity.

## Results

The results in this study focussed on the data from the extraction, isolation, characterisation and complement fixation test of acidic polysaccharides from the aerial parts of *A. mexicana.*

### Extraction and isolation of acidic polysaccharides

The aerial parts of *A. mexicana* were extracted using the ASE method as described above, where lipophilic compounds were obtained using DCM and 96% EtOH. Then 100 °C water provided the extract containing carbohydrates and low molecular weight compounds. The high molecular weight fraction of *A. mexicana* (HMAm) was separated from the low molecular weight using ultrafiltration. The HMAm fraction was dialysed, then subjected to anion exchange chromatography resulting in two polysaccharide fractions named HMAmA1 and HMAmA2.

### Carbohydrate composition of the polysaccharide fractions

The polysaccharide fractions HMAmA1 and HMAmA2 were analysed for their monomer composition, and the results are shown in Table 1. Both polysaccharide fractions contained arabinose, galactose, galacturonic acid and rhamnose in different concentrations. In HMAmA1 the major monosaccharides were galactose (15.6%) and galacturonic acid (68.4%), being more than 80% of the monomer content. In HMAmA2 the most represented monosaccharides were galactose (14.9%) and galacturonic acid (57.1%) with more than 70% of the monomer content. The presence of galacturonic acid in these two polysaccharide fractions confirmed their acidic properties.

**Table 1.** Monosaccharide compositions (mol%) of the acidic polysaccharide fractions HMAmA1 and HMAmA2.

Monosaccharide	Composition (mol%)	
	HMAmA1	HMAmA2
Arabinose	3.3	7
Rhamnose	7.8	14.5
Xylose	1.3	0
Fucose	1	0
Glucose	2.6	6.5
Galactose	15.6	14.9
Galacturonic acid	68.4	57.1
Total	100	100

### Linkage analysis of the polysaccharide fractions

In order to determine the nature of the glycosidic linkages of the different monosaccharides in the purified fractions, per-ethylation of the reduced polymers was performed, and partially *O*-ethylated alditol acetates (PEAAs) were prepared and subjected to GC-MS. The results are given in Table 2. The linkage analysis revealed the polysaccharides to be of a highly complex nature. The ratios of the linkages were calculated based on the monosaccharide composition data and the areas of the ethylated products. The main structural feature of polysaccharide fractions HMAmA1 and HMAmA2 were similar, having 1,4-linked galacturonan, with a few branch points in position 3 of galacturonic acid (GalA). These polysaccharide fractions presented a small number of terminal units of GalA. The rhamnose (Rha) units in pyranose form were mostly 1,2-linked, with branch points on position 4. The presence of Rha as terminal units was low. The low ratio of 1,2 linked Rha to 1,4-linked GalA indicated that the backbone of the polysaccharide fractions consisted of shorter rhamnogalacturonan (RG-I) structures and longer homogalacturonan regions. For both polysaccharide fractions arabinose (Ara) appeared mostly as terminal units in furanose form. The interlinkage of Ara was mostly 1,5, and all the analysed polysaccharide fractions had a small amount of 1,3,5-linked arabinose. Overall galactose (Gal) seen in pyranose form exhibited mostly terminal units and HMAmA1 showed a high amount of 1,6 and 1,3,6- linked galactose. Galactose 1,3-linked was found to be higher in fraction HMAmA2. The presence of Gal and Ara might indicate the presence of galactans, arabinans, or arabinogalactans as side chains in these polysaccharide fractions. Xylose (Xyl) and fucose (Fuc) in pyranose form, being minor monosaccharides were mainly terminal units in fraction HMAmA1.

**Table 2.** The glycosidic linkages (mol%) and derivative products of acidic polysaccharides from the aerial parts of *Argemone mexicana* were determined by reduction, ethylation and GC–MS.

Monosaccharide	Linkage type	Ratio of the different linkages %		Retention time (min)	Molar mass of primary fragments	Ethylated alditol acetates identified by GC-MS
		HMAmA1	HMAmA2			
Arabinose	T*f*	1.9	3.3	15.142	59, 132, 189	2,3,5 tri-*O*-ethyl-1,4 di-*O*-acetyl arabinitol
	1,5*f*	0.8	2.7	17.283	132, 203	2,3-di-ethyl-1,4,5-triacetyl arabinitol
	1,3,5*f*	0.5	1	18.517	132, 275	2-ethyl-1,3,4,5,tetra-*O*-acetyl-arabinitol
Rhamnose	T*p*	1.8	2.6	15.485	132, 145, 190, 203	2,3,4-tri-*O*-ethyl-1,5-di-*O*-acetyl rhamnitol
	1,2 *p*	2.3	7	17.225	145, 204	3,4-di-ethyl-1,2,5-tri-*O*-acetyl rhamnitol
	1,3 *p*	1.6	1.2	17.383	132, 145, 262	2,4-di-*O*-ethyl-1,3,5-tri-*O*-acetyl rhamnitol
	1,2,4*p*	2.1	3.7	18.7	204, 217	3-ethyl-1,2,4,5-tetra-*O*-acetyl-rhamnitol
Xylose	T*p*	1.3	0	15.717	131, 132, 189, 190	2,3,4-tri-*O*-ethyl-1,5 di-*O*-acetyl xylitol
Fucose	T*p*	1	0	16.058	132, 145, 190, 203	2,3,4-tri-*O*-ethyl-1,5-di-*O*-acetyl fucitol
Glucose	T*p*	1.6	3.1	19.633	59, 132, 189, 190, 247	2,3,4,6-tetraethyl-1,5-di-*O*-acetyl glucitol
	1,4*p*	1	3.4	21.525	59, 132, 261	2,3,6-tri-*O*-ethyl-1,4,5-tri-*O*-acetyl glucitol
Galactose	T*p*	4	3.4	19.875	59, 132, 189, 190, 247	2,3,4,6-tetraethyl-1,5-di-*O*-acetyl galactitol
	1,3*p*	3.4	7.1	21.425	59, 132, 189, 262	2,4,6-tri-*O*-ethyl-1,3,5-tri-*O*-acetyl galactitol
	1,6*p*	2.4	1.4	22.017	132, 190, 230, 261	2,3,4-tri-*O*-ethyl-1,5,6-tri-*O*-acetyl galactitol
	1,3,6*p*	5.8	3	23.65	132, 203, 262	2,4-di-*O*-ethyl-1,3,5,6-tetra-*O*-acetyl galactitol
Galacturonic acid	T*p*	4.7	4.3	19.842	61, 132, 190, 191, 249	2,3,4,6-tetraethyl-1,5-di-*O*-acetyl galactitol (D2)
	1,4*p*	59.1	48.2	21.308	61, 132, 190, 263	2,3,6-tri-*O*-ethyl-1,4,5-tri-*O*-acetyl galactitol (D2)
	1,3,4*p*	4.6	4.6	22.417	61, 132, 335	2,6-di-*O*-ethyl-1,3,4,5-tetra-*O*-acetyl galactitol (D2)

T: non-reducing terminal unit; *f*: furanose; *p*: pyranose.

### NMR spectroscopy

In order to determine whether the glycosidic linkage is in the form α or β, the polysaccharide fractions HMAmA1 and HMAmA2 were subjected to NMR spectroscopy based on their purity. The data are shown in Tables 3 and 4, respectively, for fraction HMAmA1 and fraction HMAmA2. The results revealed that fractions HMAmA1 and HMAmA2 were very similar in chemical structure and both fractions contained methyl-esters of galacturonic acid. For both fractions, galacturonic acid non-esterified also was present as repeating units (GG). Units marked EG and GE in the tables showed that the esterified and non-esterified galacturonic acids also are linked to each other. Arabinose units being terminal, 1,5- and 1,3,5-linked were present, and also 1,2- and 1,2,4 linked rhamnose, and 1,3- and 1,3,6-linked galactose. The α-linkages were identified for l-arabinose, l-rhamnose and d-galacturonic acid, while β-linkages were found for d-galactose. These data are supported by the following references: Košťálová et al. (2013), Hromádková et al. (2014), Zou et al. (2020, 2021).

**Table 3.** ^1^H and ^13^C chemical shifts (δ in ppm) of polysaccharide fraction HMAmA1 from *Argemone mexicana* aerial parts.

Linkage type	H1/C1	H2/C2	H3/C3	H4/C4	H5/C5	H6/C6	OMe	OAc
α-Araf-(1→	5.24/112.0	4.21/84.1	3.95/79.5	4.04/86.8	3.81,3.72/64.1			
α-Araf-(1→	5.14/110.0	4.22/84.1	3.95/79.5	4.08/86.9	3.81/64.0			
→5)-α-Araf-(1→	5.07/110.4	4.13/83.9	4.00/79.7	4.20/85.0	3.88/69.6			
→3,5)-α-Araf-(1→	5.10/110.4	4.27/82.2	4.09/85.1	4.13/86.8	3.80 /69.8			
→2)-α-Rhap-(1→	5.24/104.2	4.10/79.3	3.88/72.4	3.72/82.9	3.66/75.6	1.17,1.29/19.6		
→2,4)-α-Rhap-(1→	5.25/101.3	4.13/78.9		3.70/83.0	3.74/75.3	1.38/19.9		
β-Galp-(1→	4.69/106.4	3.55/73.7	3.85/84.2	4.13/71.3	3.71/78.9	3.72,3.81/64.0		
→3)-β-Galp-(1→	4.47/105.6	3.66/75.6	3.81/83.8	4.26/68.6	3.73/72.8	3.72,3.81/64.0		
→3,6)-β-Galp-(1→	4.46/106.1	3.69/77.8	3.72/82.9	4.13/71.2	3.88/76.4	4.04/72.1		
α-GalA-(1→	5.03/102.8	3.72/n.d.	3.89/72.2	4.40/81.4	4.71/74.2	177.7		
EG: −4-α-D-GalApA6Me-(1→	4.91/102.6	3.81/71.0	3.97/71.5	4.37/80.5	5.10/73.3	/173.6	3.81/55.5, 56.5	
EE: −4-α-D-GalApA6Me-(1→	4.96/102.9	3.81/71.0	3.97/71.5	4.36/80.7	5.05/73.4	/173.7		
→4-α-D-GalA	5.30/94.9	3.81/70.9	3.98/71.3	4.41/81.1	4.41/73.4	/177.6		
GG: −4-α-D-GalAp-(1→	5.08/102.0	3.73/71.0	3.97/71.7	4.42/81.0	4.69/74.2	/177.6		2.17/23.7 (O3) 2.08/22.97 (O2)
GE: −4-α-D-GalAp-(1→	5.12/102.1	3.73/71.0	3.97/71.7	4.45/81.4	4.69/74.2			

**Table 4.** ^1^H and ^13^C chemical shifts (δ in ppm) of polysaccharide fraction HMAmA2 from *Argemone mexicana* aerial parts.

Linkage type	H1/C1	H2/C2	H3/C3	H4/C4	H5/C5	H6/C6	OMe	OAc
α-Araf-(1→	5.25/112.0	4.22/84.1	3.95/79.4	4.04/86.8	3.81 3.72/64.1			
α-Araf-(1→	5.15/109.9	4.22/84.1	3.95/79.4	4.03/84.8	3.81/64.0			
→5)-α-Araf-(1→	5.08/110.4	4.13/83.8	4.00/79.6	4.20/85.0	3.88/69.5			
→3,5)-α-Araf-(1→	5.10/110.4	4.28/82.0	4.09/86.9	4.14/86.8	3.84 /69.4			
1,2 rha	5.26/101.2	4.11/79.3	3.89/72.4	3.72/82.9	3.66/75.6	1.25/19.4 1.30/19.5		
1,3 gal	4.47/105.6	3.66/75.6	3.85/84.3	4.25/68.6	3.73/72.8	3.71,3.81/64.1		
EG: −4-α-D-GalApA6Me-(1→	4.91/102.6	3.81/70.9	3.97/71.7	4.37/80.3	5.11/73.3	/173.6	3.81/55.5	
→4-α-D-GalA-(1→	5.31/95.0	3.81/70.9	3.97/71.7	4.42/80.8	4.41/73.3	/177.9		
GG: −4-α-D-GalAp-(1→	5.09/101.8	3.75/71.1	3.97/71.7	4.42/81.0	4.70/74.2	/177.9		2.10/23.1 (O2) 2.17/23.2 (O3)
1,3,4 galA	5.02/100.4	3.92/71.1	4.09/73.2	4.36/82.4	4.65/74.2	/177.1		

### Complement fixation activity

**Figure 2.**
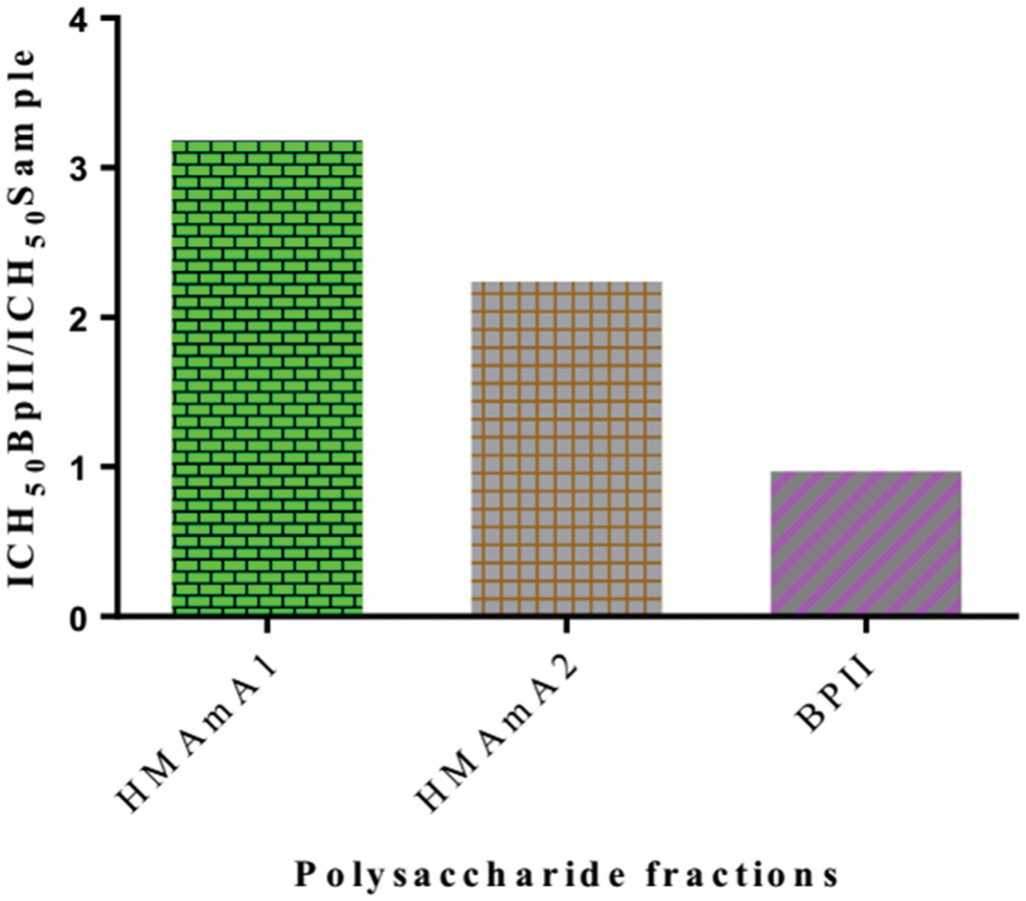
Complement fixation activity of the acidic polysaccharide fractions, given as ICH_50_ values of a polysaccharide standard BPII from *Biophytum petersianum* relative to ICH_50_ of the polysaccharide tested (ICH_50_ BPII/ICH_50_ test sample).

The acidic polysaccharide fractions exhibited a strong human complement fixation activity *in vitro* (Table 5). The activity was dose dependent and the isolated polysaccharides were more active than the positive control BPII. The concentrations of the samples giving 50% inhibition of haemolysis (ICH_50_) are shown in Table 5. Each sample was tested three times, and the ICH_50_ values are based on the means in reference to Michaelsen et al. (2000). The polysaccharide fraction HMAmA1 possessed the most potent effect, with an ICH_50_ value of 5 µg/mL. The positive control BPII showed an ICH_50_ value of 15.9 µg/mL (Figure 2). Based on the number or proportion of linkages obtained the high activity of the polysaccharide fractions could be proportional to the content in 1,6 and 1,3,6-linked galactose.

**Table 5.** ICH_50_ values (µg/mL) in the complement fixation test system by the polysaccharide fractions, indicating 50% inhibition of haemolysis.

Polysaccharide fractions	ICH_50_ (µg/mL)
HMAmA1	5
HMAmA2	7
BPII	15.9

## Discussion

Currently, bioactive phytocompounds have received great attention because of their vital health-related activities, such as antimicrobial, antioxidant, anticoagulant and antidiabetic activities, UV protection, antiviral and hypoglycaemic activities, etc. (Ullah et al. 2019). Among these components, carbohydrates known as saccharides are molecules consisting of carbon, hydrogen, and oxygen. They can also be sulphated and contain amino sugars. Carbohydrates such as monosaccharides, oligosaccharides and polysaccharides represent the most abundant biomolecules and essential components of many natural products and have attracted the attention of researchers because of their numerous human health benefits (Ruocco et al. 2016). Malian antimalarial plants contain polysaccharides (Dénou et al. 2019). From the outcomes of the polysaccharide screening on antimalarial plants used in Mali, *Argemone mexicana* was selected for deep investigations of its bioactive polysaccharides.

The defatted aerial parts were extracted with distilled water at 100 °C using an ASE apparatus. Ultrafiltration of the water extract led to a low molecular weight fraction (LMAm) which was not studied further, and a high molecular weight fraction (HMAm). Anion exchange chromatography led to the isolation of one neutral fraction, not studied further, and two acidic fractions, HMAmA1 and HMAmA2, that underwent structural characterisation and were tested for their immunomodulatory activity.

The acidic fractions contained the following monomers: arabinose (Ara), rhamnose (Rha), galactose (Gal), and galacturonic acid (Gal A) as reported in Table 1. This result confirmed the findings from the polysaccharide screening on *A. mexicana* aerial parts of our previous work (Dénou et al. 2019). Galacturonic acid is present in high amounts in HMAmA1 and HMAm2, 68.4% and 57.1%, respectively. The presence of rhamnose and galacturonic acid indicates the pectic nature of these polysaccharide fractions. The fractions contained also arabinose and galactose, indicating the presence of arabinogalactans, polymers that are commonly present in pectin as side chains on the main core. Additionally, galacturonic acid as 1,4 linked and rhamnose 1,2 (Table 2) indicate that the polymers may possess the main core consisting of a rhamnogalacturonan (indicative of RG-I) linked with longer chains of homogalacturonan as reported by Braünlich et al. (2018). Inngjerdingen and co-workers (2008) found that polysaccharide fractions from *Biophytum petersianum* possessed similar monosaccharide compositions. The amount of rhamnose, galactose, and arabinose in RG-I plays an essential role in polymer bioactivity (Inngjerdingen et al. 2008).

Additionally, the pattern of the linkages for each monosaccharide of the polymers was determined by GC-MS after ethylation and subsequent preparation of the polymers into the partly ethylated alditol acetates (Table 2). Arabinose appeared in both fractions mainly as terminal units followed by 1,5-linked units. Additionally, all the fractions also contained 1,3,5-linked Ara. Rhamnose was present in all the fractions as terminal units and also 1,2 and 1,2,4 linked units. A small amount of 1,3 linked units were found in both fractions. Xylose was found as terminal units except for HMAmA2. Fucose was present as terminal units in HMAmA1. Galactose appeared in both fractions as terminal units and also as 1,3; 1,6 and 1,3,6-linked units. The presence of 1,3-linked and 1,3,6-linked Gal indicate arabinogalactan II (AG-II) structures in the fractions. Galacturonic acid appeared in both polymers, mainly as 1,4 linked followed by 1,3,4 linked units. Galacturonic acid was also found at the high amount in both polymers. These acidic polysaccharides could be pectins containing ‘smooth’ regions, consisting of homogalacturonans and ‘hairy’ regions, consisting of a rhamnogalacturonan backbone carrying side chains, typically 1,5-linked arabinans or 1,3-linked galactans. The presence of 1,6 and 1,3,6 linked galactose and terminal arabinose could explain the presence of arabinogalactan II (AGII) (Togola et al. 2008). At least two ethylated alditols from each monomer were identified at different retention times (Table 2). To our knowledge, this is the first investigation of the glycosidic linkages and derivative products on polysaccharides from *A. mexicana.* Therefore, this result could be a reference for other works in the future.

The polysaccharide fractions HMAmA1 and HMAmA2 were also characterised by 1 D and 2 D NMR spectroscopy and compared with chemical shift values from published results (Zou et al. 2020, 2021). From Tables 3 and 4, it can be seen that assignments can be given for most of the structural details that also was found for the methylation studies. The signals from NMR for both fractions showed that methyl esters of galacturonic acid were present as neighbouring units (EE), as also found by (Košťálová et al. 2013; Hromádková et al. 2014), Galacturonic acids not esterified were also present as repeating units (GG). Units marked EG and GE in the tables showed that the esterified and not esterified galacturonic acids also are linked to each other. Arabinose units being terminal, 1,5- and 1,3,5-linked are verified, and so are 1,2- and 1,2,4 linked rhamnose and 1,3- and 1.3.6 linked galactose. α-Linkages are confirmed for l-arabinose, l-rhamnose and d-galacturonic acid, while β-linkages are found for d-galactose. From a similar work on *Platycodon grandiflorus*, the result of **^1^**H, ^13^C, and 2 D NMR spectra revealed typical signals belonging to α-1,4-GalpA, β-1,4-linked Galp, and 1,3,5-linked Araf, demonstrating a pectin polysaccharide with highly branched 1,5-α-l-arabinan and 1,4-β-d-galactan presented in PGP-I-I (Zou et al. 2021).

The complement system plays an important role in the immune defense, such as primary defense against bacterial invasions and viral infections. Complement fixating activity by polysaccharides from plants has previously been shown as an indicator of effects on the immune system (Michaelsen et al. 2000; Inngjerdingen et al. 2013; Zou et al. 2014). Recently, scientists established the effect of the complement system on parasites including *Plasmodium falciparum* (Kurtovic et al. 2020). The acidic polysaccharides (HMAmA1, and HMAmA2) fractionated from 100 °C water extract of *A. mexicana* aerial parts were tested *in vitro* on the complement system. Data revealed that both polymers showed complement fixation activity. The acidic polysaccharide HMAmA1 exhibited the highest activity with an ICH_50_ value of 5 µg/mL while the positive control BPII had an ICH_50_ value of 15.9 µg/mL (Table 5). These values are about three times higher compared to the positive control (BPII) (Figure 2). This fact could partly be due to their molecular weights which were not determined in this study. Previous work has shown that acidic polysaccharides with high molecular weights exhibited the most potent activities (Zou et al. 2015). In addition, these same authors found that pectin with low molecular weight and a highly branched structure can have a high complement fixation activity (Zou et al. 2015). On the other hand, RG-I and arabinogalactans could also play an important role in the complement fixation activity (Togola et al. 2008).

## Conclusions

This current work reveals for the first time that a 100 °C aqueous extract of aerial parts of *A. mexicana* contains acidic polysaccharides with an immunomodulatory effect. This could be used to boost the immune defense, and thus useful as a supportive treatment in infectious diseases like malaria.

## References

[CIT0001] Austarheim I, Christensen BE, Hegna IK, Petersen BO, Duus JO, Bye R, Michaelsen TE, Diallo D, Inngjerdingen M, Paulsen BS. 2012. Chemical and biological characterization of pectin-like polysaccharides from the bark of the Malian medicinal tree *Cola cordifolia*. Carbohydr Polym. 89(1):259–268. 24750632 10.1016/j.carbpol.2012.03.005

[CIT0002] Barsett H, Paulsen BS, Habte Y. 1992. Further characterization of polysaccharides in seeds from *Ulmus glabra* Huds. Carbohydr Polym. 18(2):125–130.

[CIT0003] Braünlich PM, Inngjerdingen KT, Inngjerdingen M, Johnson Q, Paulsen BS, Mabusela W. 2018. Polysaccharides from the South African medicinal plant *Artemisia afra*: structure and activity studies. Fitoterapia. 124:182–187. 29155274 10.1016/j.fitote.2017.11.016

[CIT0004] Cazón P, Velazquez G, Ramírez JA, Vázquez M. 2017. Polysaccharide-based films and coatings for food packaging: a review. Food Hydrocoll. 68:136–148.

[CIT0005] Chambers RE, Clamp JR. 1971. An assessment of methanolysis and other factors used in the analysis of carbohydrate-containing materials. Biochem J. 125(4):1009–1018. 5144210 10.1042/bj1251009 PMC1178263

[CIT0006] Copeland L, Blazek J, Salman H, Tang MC. 2009. Form and functionality of starch. Food Hydrocoll. 23(6):1527–1534.

[CIT0007] Cui SW. 2005. Ed. Food carbohydrates: chemistry, physical properties, and applications. Boca Raton (FL): CRC Press.

[CIT0008] Dénou A, Ahmed A, Dafam DG, Ochala SO, Omale S, Inngjerdingen KT, Sanogo R, Diallo D, Paulsen BS, Aguiyi JC. 2020b. Safety evaluation of polysaccharides isolated from the water extract of *Argemone mexicana* L. (Papaveraceae) in *Drosophila melanogaster*. J Appl Pharm Sci. 10(02):044–048.

[CIT0009] Dénou A, Ahmed A, Dafam DG, Yakubu TP, Sanogo R, Diallo D, Alemika TE. 2020a. Pharmacognostic, physicochemical and phytochemical investigations on aerial parts of *Argemone mexicana* L. Res J Pharmacogn. 7(3):15–24.

[CIT0010] Dénou A, Togola A, Inngjerdingen KT, Zhang B-Z, Ahmed A, Dafam DG, Aguiyi JC, Sanogo R, Diallo D, Paulsen BS. 2019. Immunomodulatory activities of polysaccharides isolated from plants used as antimalarial in Mali. J. Pharmacognosy Phytother. 11(3):35–42.

[CIT0011] Diallo D, Diakité C, Mounkoro PP, Sangaré D, Graz B, Falquet J, Giani S. 2007. The management of malaria by traditional healers in the health areas of Kende (Bandiagara) and Finkolo (Sikasso) in Mali. Mali Med. 12:1–8. 19434974

[CIT0012] DuBois M, Gilles KA, Hamilton JK, Rebers PA, Smith F. 1956. Colorimetric method for determination of sugars and related substances. Anal Chem. 28(3):350–356.

[CIT0013] Grønhaug TE, Ghildyal P, Barsett H, Michaelsen TE, Morris G, Diallo D, Inngjerdingen M, Paulsen BS. 2010. Bioactive arabinogalactans from the leaves of *Opilia celtidifolia* Endl. ex Walp. (Opiliaceae). Glycobiology. 20(12):1654–1664. 20729344 10.1093/glycob/cwq120

[CIT0014] Grønhaug TE, Kiyohara H, Sveaass A, Diallo D, Yamada H, Paulsen BS. 2011. Beta-D-(1→4)-galactan-containing side chains in RG-I regions of pectic polysaccharides from Biophytum petersianum Klotzsch. contribute to expression of immunomodulating activity against intestinal Peyer's patch cells and macrophages. Phytochemistry. 72(17):2139–2147. 21880338 10.1016/j.phytochem.2011.08.011

[CIT0015] Hromádková Z, Koštálová Z, Vrchotová N, Ebringerová A. 2014. Non-cellulosic polysaccharides from the leaves of small balsam (*Impatiens parviflora* DC.). Carbohydr Res. 389:147–153. 24680545 10.1016/j.carres.2014.01.016

[CIT0016] Husna SA, Reddy VJ. 2017. A review on *Argemone mexicana*. Int J Pharmacol Res. 7:170–174.

[CIT0017] Ibrahim HA, Umar MA, Bello BA, Aliyu A, Ahmad A. 2016. Analgesic and anti-inflammatory studies on the roots *Argemone mexicana* Linn (Family: Papaveraceae). J Pharm Biol Sci. 11(3):92–95.

[CIT0018] Inngjerdingen KT, Ballo N, Zhang B, Malterud KE, Michaelsen TE, Diallo D, Paulsen BS. 2013. A comparison of bioactive aqueous extracts and polysaccharide fractions from roots of wild and cultivated *Cochlospermum tinctorium* A. rich. Phytochemistry. 93:136–143. 23582214 10.1016/j.phytochem.2013.03.012

[CIT0019] Inngjerdingen M, Inngjerdingen KT, Patel TR, Allen S, Chen X, Rolstad B, Morris GA, Harding SE, Michaelsen TE, Diallo D, et al. 2008. Pectic polysaccharides from *Biophytum petersianum* Klotzsch, and their activation of macrophages and dendritic cells. Glycobiology. 18(12):1074–1084. 18809620 10.1093/glycob/cwn090

[CIT0020] Kim JB, Carpita NC. 1992. Changes in esterification of the Uronic acid groups of cell wall polysaccharides during elongation of maize coleoptiles. Plant Physiol. 98(2):646–653. 16668690 10.1104/pp.98.2.646 PMC1080239

[CIT0021] Košťálová Z, Hromádková Z, Ebringerová A. 2013. Structural diversity of pectins isolated from the styrian oil-pumpkin (*Cucurbita pepo* var. *styriaca*) fruit. Carbohydr Polym. 93(1):163–171. 23465915 10.1016/j.carbpol.2012.05.017

[CIT0022] Kurtovic L, Boyle MJ, Opi DH, Kennedy AT, Tham WH, Reiling L, Chan JA, Beeson JG. 2020. Complement in malaria immunity and vaccines. Immunol Rev. 293(1):38–56. 31556468 10.1111/imr.12802 PMC6972673

[CIT0023] Michaelsen TE, Gilje A, Samuelsen AB, Hogasen K, Paulsen BS. 2000. Interaction between human complement and a pectin type polysaccharide fraction, PMII, from the leaves of *Plantago major* L. Scand J Immunol. 52(5):483–490. 11119247 10.1046/j.1365-3083.2000.00801.x

[CIT0024] Nemzer BV, Kalita D, Yashin AY, Nifantie NE, Yashin YI. 2019. *In vitro* antioxidant activities of natural polysaccharides: an overview. JFR. 8(6):78–93.

[CIT0025] Pathak R, Goel A, Tripathi SC. 2021. Medicinal property and ethnopharmacological activities of *Argemone mexicana*: an overview. Ann Rom Soc Cell Biol. 25:1615–1641.

[CIT0026] Paulsen BS, Barsett H. 2005. Bioactive pectic polysaccharides. Adv Polym Sci. 186:69–101.

[CIT0027] Ray P, Chatterjee S, Saha P. 2020. Immunomodulatory activity of natural polysaccharides in combating Covid-19, cancer, inflammatory disorders: a review. Int J Life Sci Pharma Res. 10:191–206.

[CIT0028] Ruocco N, Costantini S, Guariniello S, Costantini M. 2016. Polysaccharides from the marine environment with pharmacological, cosmeceutical and nutraceutical potential. Molecules. 21(5):551–516. 27128892 10.3390/molecules21050551 PMC6273702

[CIT0029] Sanogo R, Doucouré M, Fabre A, Haïdara M, Diarra B, Dénou A, Kanadjigui F, Benoit VF, Diallo D. 2014. Standardization and industrial production test of an antimalarial syrup based on extracts of *Argemone mexicana* L. Revue CAMES – Série *Pharm*. Méd Trad Afr. 17(1):15–20.

[CIT0030] Sharanappa R, Vidyasagar GM. 2014. Plant profile, phytochemistry and pharmacology of *Argemone mexicana* Linn. A review. Int J Pharm Pharm Sci. 6(7):45–53.

[CIT0031] Sweet DP, Shapir RH, Albersheim P. 1975. Quantitative analysis by various GLC response factor theories for partially methylated and partially ethylated alditol acetates. Carbohydr Res. 40(2):217–225.

[CIT0032] Thorat VH, Ghorpade S. 2018. *Argemone mexicana* Linn. having medicinal property: review. Int J Pharmacogn. 5(2):82–90.

[CIT0033] Togola A, Inngjerdingen M, Diallo D, Barsett H, Rolstad B, Michaelsen TE, Paulsen BS. 2008. Polysaccharides with complement fixing and macrophage stimulation activity from *Opilia celtidifolia*, isolation and partial characterization. J Ethnopharmacol. 115(3):423–431. 18053663 10.1016/j.jep.2007.10.017

[CIT0034] Ullah S, Khalil AA, Shaukat F, Song Y. 2019. Sources, extraction and biomedical properties of polysaccharides. Foods. 8(8):304–323. 31374889 10.3390/foods8080304 PMC6723881

[CIT0035] Wang H, Liu YM, Qi ZM, Wang SY, Liu SX, Li X, Wang HJ, Xia XC. 2013. An overview on natural polysaccharides with antioxidant properties. Curr Med Chem. 20(23):2899–2913. 23627941 10.2174/0929867311320230006

[CIT0036] Wang J, Hu S, Nie S, Yu Q, Xie M. 2016. Reviews on mechanisms of *in vitr*o antioxidant activity of polysaccharides. Oxid Med Cell Longev. 2016:5692852–5692813. 26682009 10.1155/2016/5692852 PMC4670676

[CIT0037] Yamada H, Kiyohara H. 1999. Complement-activating polysaccharides from medicinal herbs. In Wagner, H. (Ed.), Immunomodulatory agents from plants. Basel: Birkhäuser; p. 161–202.

[CIT0038] Yang L, Zhang L. 2009. Chemical structural and chain conformational characterization of some bioactive polysaccharides isolated from natural sources. Carbohydr. Polym. 76(3):349–361.

[CIT0039] Yu Y, Shen M, Song Q, Xie J. 2018. Biological activities and pharmaceutical applications of polysaccharide from natural resources: a review. Carbohydr Polym. 183:91–101. 29352896 10.1016/j.carbpol.2017.12.009

[CIT0040] Zou YF, Barsett H, Ho GTT, Inngjerdingen KT, Diallo D, Michaelsen TE, Paulsen BS. 2015. Immunomodulating pectins from root bark, stem bark, and leaves of the malian medicinal tree *Terminalia macroptera*, structure activity relations. Carbohydr Res. 403:167–173. 24909378 10.1016/j.carres.2014.05.004

[CIT0041] Zou YF, Chen M, Fu YP, Zhu ZK, Zhang YY, Paulsen BS, Rise F, Chen Y-L, Yang Y-Z, Jia R-Y, et al. 2021. Characterization of an antioxidant pectic polysaccharide from *Platycodon grandiflorus*. Int J Biol Macromol. 175:473–480. 33571586 10.1016/j.ijbiomac.2021.02.041

[CIT0042] Zou YF, Zhang BZ, Barsett H, Inngjerdingen KT, Diallo D, Michaelsen TE, Paulsen BS. 2014. Complement fixing polysaccharides from *Terminalia macroptera* root bark, stem bark and leaves. Molecules. 19(6):7440–7458. 24914893 10.3390/molecules19067440 PMC6270672

[CIT0043] Zou Y-F, Zhang Y-Y, Paulsen BS, Rise F, Chen Z-L, Jia R-Y, Li L-X, Song X, Feng B, Tang H-Q, et al. 2020. Structural features of pectic polysaccharides from stems of two species of *Radix Codonopsis* and their antioxidant activities. Int J Biol Macromol. 159:704–713. 32422266 10.1016/j.ijbiomac.2020.05.083

